# Automatic Focus Assessment on Dermoscopic Images Acquired with Smartphones

**DOI:** 10.3390/s19224957

**Published:** 2019-11-14

**Authors:** José Alves, Dinis Moreira, Pedro Alves, Luís Rosado, Maria João M. Vasconcelos

**Affiliations:** Fraunhofer Portugal AICOS, 4200-135 Porto, Portugal

**Keywords:** mobile dermatology, image acquisition, image quality assessment, feature extraction, machine learning

## Abstract

Over recent years, there has been an increase in popularity of the acquisition of dermoscopic skin lesion images using mobile devices, more specifically using the smartphone camera. The demand for self-care and telemedicine solutions requires suitable methods to guide and evaluate the acquired images’ quality in order to improve the monitoring of skin lesions. In this work, a system for automated focus assessment of dermoscopic images was developed using a feature-based machine learning approach. The system was designed to guide the user throughout the acquisition process by means of a preview image validation approach that included artifact detection and focus validation, followed by the image quality assessment of the acquired picture. This paper also introduces two different datasets, dermoscopic skin lesions and artifacts, which were collected using different mobile devices to develop and test the system. The best model for automatic preview assessment attained an overall accuracy of 77.9% while focus assessment of the acquired picture reached a global accuracy of 86.2%. These findings were validated by implementing the proposed methodology within an android application, demonstrating promising results as well as the viability of the proposed solution in a real life scenario.

## 1. Introduction

Malignant melanoma is the 19th most common cancer among men and women, with nearly 300,000 new cases in 2018 while non-melanoma skin cancer is the 5th most common cancer, with over 1 million diagnoses worldwide in 2018, a number which is considered to be an underestimation [1,2]. The increasing incidence of melanoma and the potential risk for misdiagnosis make the management of melanocytic lesions particularly challenging for both dermatologists and primary care physicians [3], resulting in a considerable economic burden for public health services [2,4]. However, if detected early, the success rates of successfully treating this type of cancer are very high, therefore the development of methodologies to aid the monitoring processes and assisting diagnosis are of high importance.

Dermatology is the branch of medicine dealing with the diagnosis, treatment and prevention of skin diseases. Acquisition of dermoscopic images of skin lesions and melanocytic lesions is a standard procedure in dermatological practice, which results in a valuable asset for every clinician [5]. By definition, dermoscopy is a non-invasive skin imaging technique which uses a dermatoscope with optical magnification and polarized lighting to highlight submacroscopical structures, making them visible to the naked eye [5]. The term melanocytic lesion refers to proliferations of neural crest derived melanocytic cells (which produce the dark pigment in the skin) ranging from benign freckles and nevi to malignant melanoma [6].

The recent advances in mobile health (m-Health), a rising digital health sector that provides healthcare support, delivery and intervention via mobile technologies such as smartphones, led to an increase in the number of available self-care and telemedicine solutions related to the skin. Early detection, surveillance, easier access to health care services, avoiding unnecessary medical appointments or just documenting specific cases, are some of the reasons behind the creation of such systems, particularly in the dermatology field [7,8,9,10,11]. However, in order for the specialists to be able to provide reliable diagnosis, it is essential for them to receive standardized information and guarantee its quality, especially when dealing with clinical images.

This context motivated the development of a new algorithm for automated focus assessment of dermoscopic images, more specifically, by correctly identifying structures of interest, that is, skin moles, while simultaneously assessing the quality of those images. Particularly, an image focus validation approach was developed to perform real-time image quality control. With this work, we aim to contribute to the standardization of image acquisition in dermoscopy via mobile devices, by assisting and guiding the user during the acquisition process of skin lesions, and consequently facilitate both monitoring and diagnosis procedures.

This paper is structured as follows: Section 1 presents the motivation and objectives of this work; Section 2 presents the related work; Section 3 provides an overview of the system architecture along with the datasets description and the methodology used; in Section 4 and Section 5 the results and discussion are presented; Section 6 highlights the main conclusions of this study and points out possible directions for future work.

## 2. Related Work

Due to the most recent technological breakthroughs in the area, a new generation of mobile devices has appeared. These devices, such as smartphones, are typically low cost, light weight, portable, and have high computational power, thus, constituting one of the most common forms of image acquisition and processing. Especially in dermatology, where these types of devices are being used to acquire skin lesion images and exchange information among general practitioners, dermatologists or patients [12,13]. Acquisition of these images is becoming more and more frequent, with a large number of images being captured every day for documenting clinical findings, self reporting or even for educational or research purposes [5].

As can be seen in the recent literature, a wide variety of m-Health solutions are available [12,13]. These solutions can be as simple as an smartphone application for acquiring and transferring skin lesion images to a dermatologist (SAF) or more complex, as the fully automated solutions in which a diagnostic is presented after the picture is taken [7,8,9,14]. However, these solutions generally produce different diagnosis in comparison to the dermatologists decisions [15], and often do not provide guidance during the image acquisition process nor do they assess the quality of the final picture [8,9,10,12].

Recent studies have studied the influence of several conditions, such as lighting, background color, field of view, image orientation, focus and depth of field, resolution, scale, color calibration and image storage may have on the acquired image as being key aspects in dermatology [16,17]. Thus, the image quality assessment (IQA) of newly acquired skin images should be a mandatory step [12,17,18,19]. Especially when using smartphones’ built-in cameras, the real-time evaluation of those images should be addressed, not only to assist or guide the acquisition process, but also to simultaneously ensure that no additional artifacts, such as motion and defocus blur, will be present in the final acquired image [18,19]. From the available solutions, only the SkinVision App [7] includes an algorithm to assess the quality of the picture and states that it reduces the number of blurry photos by about 52% [20]. Therefore, to the best of the authors’ knowledge, image quality assessment is generally performed only by suggesting a couple of best practices and/or by reporting the environment and used camera settings during the acquisition [8,9,11].

In terms of IQA objective methods, the literature is quite vast and the used methods can be divided in three different groups—full reference methods, where a reference image is present; reduced reference methods, where only partial information about the original image is available together with a set of discriminative features; no reference methods, where no original image is available [21]. For the no-reference image quality metrics, a different types of metrics have been proposed in recent years—distortion specific (e.g., specific type of blur, uneven illumination, etc.) and learning based on natural scene statistics metrics are some of the categories these metrics may fall into [22]. Also transform-based, statistics, directional or geometric based features are some metrics that are widely used to discriminate the quality of an image where no reference image is provided [23]. In Reference [24] the authors have explored a no reference methodology for uneven illumination assessment of 30 dermoscopic images with different degrees of real uneven illumination. The authors obtained 0.902 and 0.895 for Pearson linear correlation coefficient (LCC) and Spearman rank-order correlation coefficient (SROCC) respectively, when comparing predicted results to the subjective ground truth annotations. Both LCC and SROCC are between 0 and 1, where values close to 1 indicate a better performance. In a later work [25], the same group of researchers used a similar approach in order to assess two different types of distortions in an image, the uneven illumination and blur, reporting 0.841 and 0.859 for LCC and SROCC, respectively, when analyzing 162 images suffering from real distortions.

## 3. System Architecture

The proposed system allows the focus assessment of skin moles in dermoscopic images by using a feature-based machine learning methodology. The system was designed to guide the user throughout the acquisition process by means of an image focus validation approach, followed by the IQA of the acquired picture.

**Figure 1:**
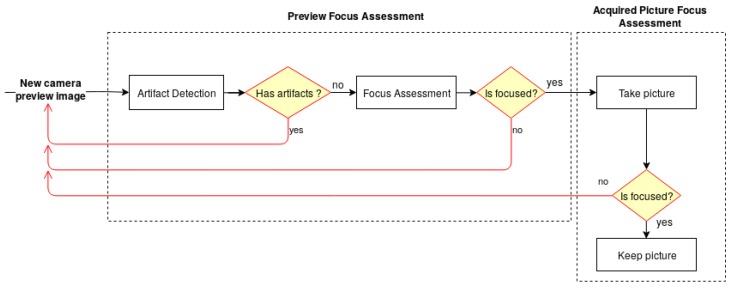
Diagram of the system architecture for the automatic focus assessment on skin lesion dermoscopic images acquired with smartphones.

The architecture of the developed solution, illustrated in Figure 1, is divided into two main modules—the Preview Focus Assessment and the Acquired Picture Focus Assessment. For each frame obtained from the camera preview, the preview focus assessment methodology performs a preliminary verification for artifact detection, followed by the skin mole focus assessment of the preview image. This step is particularly important to guarantee that the smartphone is focusing on a skin mole and not on the artifacts that the lens may contain. Once the preview image passes the first verification module, the user receives an indication that the preview image is focused and is able to proceed with the image acquisition. The preview focus assessment process is repeated for each upcoming frame from the camera preview providing this feedback continuously to the user in real time through the application interface. Afterwards, in the acquired picture focus assessment module, the dermoscopic image is evaluated again in terms of quality and presented to the user immediately after an acquisition.

### 3.1. Datasets

The present work aims to assist the acquisition process of dermoscopic images acquired with smartphones by providing automatic focus assessment. Some public databases of skin lesions contain dermoscopic images that were classified by dermatologists for diagnosis purposes, such as ISIC, PH2 or HAM10000 [26]. These databases contain images with sufficient quality for clinical decision making, since the low quality images are usually discarded. Moreover, for each skin lesion there is only one image and generally no additional information regarding the device used in the acquisition is provided. Therefore, to the best of our knowledge, there is no publicly available image quality database that includes dermoscopic images of skin lesions with a different level of focus, so in the scope of this work two new datasets were collected. A skin lesion dataset of dermoscopic images, focused and non-focused, was gathered including both images from the smartphone camera (preview and acquired pictures). An additional dataset composed by dermoscopic images with lens artifacts and different backgrounds was collected to design the preview focus assessment algorithm.

For the collection of both datasets, two different dermoscopes were used—Dermlite DL1 (DL1) [27] and Dermlite DL3 (DL3) [28]. Both of the chosen dermoscopes in this study can be used with a different range of smartphones and allow standalone usability. The price of the considered devices ranges between 360 and 850 euros at the time of this study. Table 1 shows the specifications of the devices and in Reference [29] a more detailed study on their differences in terms of color reproduction, image area and distortion, illumination, sharpness and differential structures visibility is presented.

Regarding the variance between the acquisition devices, 11 different smartphones with different cameras properties were used during the acquisition. The overall robustness of the proposed solution across different smartphones was addressed in this approach, by including the highest possible number of different devices in the study together with the use of two different dermoscopes. A complete list of the mobile devices used alongside with the major characteristics is available in Table 2.

#### 3.1.1. *DermIQA* Dataset

In order to address the problem of assessing image quality and focus of skin moles images in real-time, a dataset of focused and non-focused images was collected, named the Dermoscopic Image Quality Assessement (*DermIQA*) dataset. This dataset is composed of a total of 1979 images of skin moles from 14 different Caucasian subjects. The images were acquired using the aforementioned dermoscopes together with the 11 different smartphones. The goal of collecting this dataset is to have at least one blurred and one focused image for each skin mole, smartphone and dermoscope. For each acquisition both camera preview image and captured image were saved for the following reasons: (i) in the preview stage the goal is to assess the image in terms of image stabilization and standardization, where as in the acquired image the goal is to check the quality of the image that will be saved in the system and used for monitoring or diagnosis purposes; (ii) also, the preview image has smaller resolution than the acquired images (720 × 1280 vs. 1080 × 1920 px). A summary of the number of collected images and their distribution regarding the focus level is provided in Table 3.

**Figure 2:**
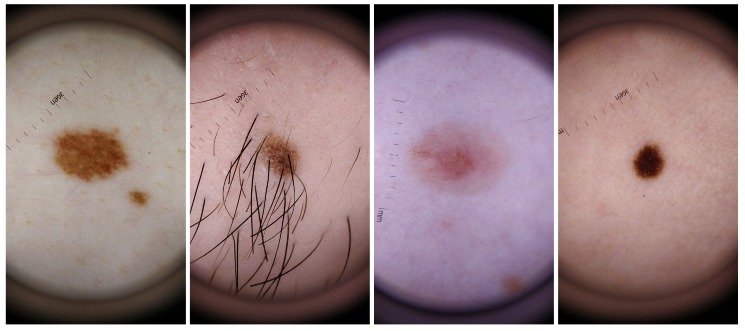
Illustrative examples of skin mole present in the *DermIQA* dataset.

For the dataset collection, different aspects where taken into consideration in order to guarantee the most variability possible within the recruited voluntary participants. Skin lesions were acquired from subjects with different genders and skin tones, with phototypes varying from I to V. Also, the skin lesions selected had different colors, sizes and shapes as well as presence/absence of hairs or beard. The inclusion of this variability in the dataset aimed also to select features that were able to deal with this variability and therefore more robust and suitable to be used in real life scenario. For a better characterization of this specific dataset, complementary information about the size, shape, border type, color and absence or not of hair for each analyzed skin mole is presented in Table 4, whereas in Figure 2 illustrative examples of skin lesions are depicted.

#### 3.1.2. *DermArtifacts* Dataset

While conducting preliminary experiments, it was observed that some images with no useful information and/or with only lenses artifacts were classified as focused images. Therefore, there was the need to construct a dataset that had both relevant structures as skin moles (with both focused and unfocused examples) and non-relevant structures like artifacts, which served as a basis for building the Artifact Detection module referred previously.

**Figure 3:**
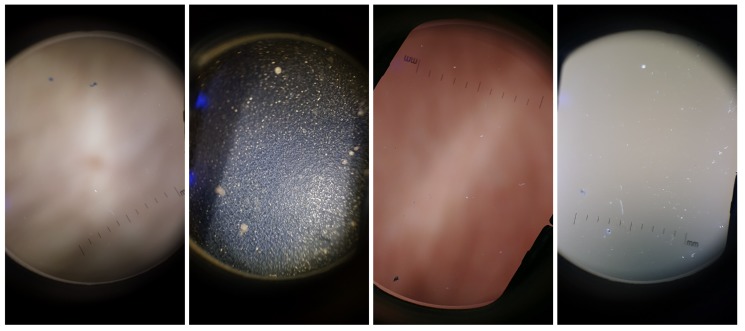
Illustrative examples of background and artifact images present in the *DermArtifacts* dataset.

The Dermoscopic Lens Artifacts (*DermArtifacts*) image dataset is composed of a total of 232 camera preview images—131 skin moles images and 131 images with different backgrounds and/or with lenses artifacts, representing the negative class of interest. The skin mole images were arbitrarily chosen from the *DermIQA* dataset, whereas the 131 images with artifacts were additionally collected. Some of the smartphones from the ones listed in the Table 2 was used to collect those images with different backgrounds and lens artifacts, since there is less variability in the images (Samsung J5, S6, S7, S8, LG G6, Motorola G5, Nexus 5X and OnePlus 5). Representative examples of lens artifact images are presented in Figure 3.

It should be noted that all images on *DermArtifacts* and *DermIQA* datasets were annotated by the authors, no dermatology specialist was consulted, and are therefore more prone to human error and subjectivity of the labelling process.

### 3.2. Image Focus Assessment Pipeline

A feature-based machine learning approach was used in order to develop an image focus assessment algorithm. The approach followed the usual machine learning pipeline, including feature extraction, model training and validation, as is described in the following subsection. Additionally, as the proposed system is intended to run in real-time in a wide range of mobile devices, it is expected that some models might have limited computational resources. Therefore, this limitation greatly influenced the design of the machine learning pipeline, particularly in terms of giving major focus to the usage of lightweight image quality features, as well as selecting a computationally suitable machine learning classifier.

#### 3.2.1. Feature Extraction

The first step of this pipeline is the extraction of several state-of-the-art image quality related features. Each image was primarily cropped to a central square with the size of 70% of the original image, not only due to processing constraints, but also to remove non-interest regions from the original images (e.g., the black regions near the borders caused by the dermoscopy device). This square region is used by the algorithm to extract the metrics and make decisions accordingly. The square image is then converted to the gray scale colorspace $I_{Gray}$, and a new image $I_{Blur}$ is generated by applying a mean filter to the gray scale image. The kernel size $kernel_{Size}$ used to create $I_{Blur}$ is calculated according to the following equation:

(1) $$kernel_{Size}=\left\{\begin{array}{cc} \frac{min(I_{Gray}^{Width},I_{Gray}^{Height})}{75}, & \mathrm{if}\;\frac{min(I_{Gray}^{Width},I_{Gray}^{Height})}{75}=odd\\ \frac{min(I_{Gray}^{Width},I_{Gray}^{Height})}{75}+1, & \mathrm{otherwise} \end{array}\right.$$

The generation of $I_{Blur}$ image is important due to the fact that a blurred image usually has soft edges, less color variation and brightness, meaning that the pixels of the same area of the image will have, in the grayscale image, similar color values, thus resulting in a smaller variance of the color values. Therefore, the impact of filtering an already blurred image, which has similar color values around each pixel, will be significantly smaller than when applied to a non blurred one. Afterwards, several image features for assessing blur distortion were extracted for both $I_{Gray}$ and $I_{Blur}$ images. The complete set of the considered focus metrics was already reported in a previous study [23], it being possible to categorize them into five broad groups according to their working principles—Gradient based, Laplacian based, Statistical based, Discrete Cosine Transform (DCT)/Discrete Fourier Transform (DFT) based and Other principles (see Table 5 for a detailed summary).

Additionally, it should be noted that the magnitude of the absolute value of $I_{Gray}$ and $I_{Blur}$ focus metrics greatly depends on the specific characteristics of each skin mole (e.g., texture, edges, etc.). So in order to achieve an adaptive approach that effectively generalizes for different image characteristics, we added a new subset of features based on relative values, that is, it consists more specifically in the difference and the quotient between the focus metrics values of $I_{Gray}$ and $I_{Blur}$. It should be noted that the inclusion of similar relative focus features using artificially blurred images was already explored for other use cases with very promising results, including microscopic [30] and skin wounds [31] images. When computing the relative features, its values will be smaller for the blurred images due to the lower variation of the gray color values between both forced blurred and original images. By merging all the extracted absolute and relative focus features, we obtained a feature space with a total of 360 metrics.

#### 3.2.2. Models Training and Optimization

Following the system architecture diagram, the aim of this work was to find accurate and robust models for three different tasks—(i) artifact detection, (ii) preview focus assessment and iii) acquired picture focus assessment.

In order to train the different models, two datasets were collected (as explained in the previous section), which were then subdivided into train and test datasets according to each correspondent classification task. For the *DermArtifact* dataset, this division was performed by keeping 70% and 30% of the data as train and test sets, respectively. As for the *DermIQA* dataset, this division was performed by keeping all the images from 9 subjects as the train set and images from 5 subjects as the test set, with the purpose of having a wide variety of skin moles both in the training and test set. Since with a random split, the test set could end up with more than two or three similar skin moles, and since the objective is to validate the algorithm by using moles with the highest variety of characteristics as possible, these moles should also be represented in the test set in order to ensure a more general and robust outcome. Also, this division was made for both preview and acquired images separately, enabling the creation of two different classification models, one for assessing only the preview images (ii) and another to assess the final acquired images (iii).

Due to the limited computational capabilities of some smartphone models, and in order to ensure not only real-time computational calculation of focus metrics but also real-time feedback to the user regarding the focus level on camera preview frames, we opted to use a Decision Tree classifier. Furthermore, the subset of values for hyper-parameter optimization used for model optimization during training are further detailed in Table 6. All models were trained on a desktop, based on the implementation included in the Scikit-learn Python module [32].

Moreover, and for each aforementioned classification task, the following optimization pipeline was adopted to train and select the best overall model:

1. Run grid search with the previously defined model hyper-parameters using the stratified cross validation technique (10-fold cross validation). *F1-Score* or *Informedness/Youden Index* metric was used as the classification metric to optimize when evaluating the *DermArtifacts* or *DermIQA* datasets, respectively;
2. Take the best estimator chosen by the search in (1) and evaluate its performance on the correspondent test set;
3. Keep best estimator parameters if the chosen metric to optimize for on train set is greater than the previously saved/stored one and its *Recall* value on test set is greater than 85%;
4. Repeat the above steps for 5000 iterations.

## 4. Results

### 4.1. Artifact Detection Results

The Artifact Detection module in our proposed pipeline was created using the *DermArtifacts* dataset. The optimized model found in the training phase, together with its respective best selected features, was assessed on the correspondent test set data, recurring to classification metrics as Accuracy, Recall, Precision, Specificity and F1-Score. The best classification results for this task are presented in Table 7 (first row), and are computed using only two different features, namely the Difference Between the Sum of Values of the Grey Level Variance of the gray and blurred image ($GLVA_{I_{Gray}}^{SUM}-GLVA_{I_{Blur}}^{SUM}$) and the Sum of the Perceptual blur metric of the blurred image on the y direction ($PRCB_{I_{Blur}}^{SUM_{Y}}$). Thus, as one can infer from these results, an overall accuracy of 97.3% was achieved for the detection of artifacts and non-interest structures on preview camera images.

### 4.2. Preview Images Focus Assessment Results

Assessment of the images from the camera preview is perhaps the most important step within the proposed pipeline, since it is on this stage that the most valuable information can be provided to the user in real-time. Thus, making this feedback important for assisting the process of acquiring focused images of a skin mole. Classification results of the *Focus Assessment* module on the test set of the *DermIQA* dataset are presented in the Table 7 (second row). The best model obtained consists in the usage two features, namely the *Division between the Sum of the values of the Image Curvature of blur and gray image* ($CURV_{I_{Blur}}^{SUM}/CURV_{I_{Gray}}^{SUM}$) and the *Maximum of the Variance of Laplace of the gray image* ($LAPV_{I_{Gray}}^{MAX}$). As it can be seen in the Table 7, an overall accuracy of 83.7% was attained for correctly identifying if a certain preview image is focused or not.

As the Preview Focus Assessment module is composed by two blocks, namely Artifact Detection and Focus Assessment modules, the combined performance also needed to be addressed. Thus, these two models that first evaluate artifact presence followed by focus of camera preview images were tested with all the preview images present in the *DermIQA* dataset, being the results presented in the Table 7 (third row). As it can be seen from this results, an overall accuracy of 77.9% is achieved, which is less than those obtained when using the Artifact or Focus Assessment models alone. Despite this decrease in the overall performance due to this trade-off, relatively accurate results in terms of camera preview assessment are obtained when only using a total of 4 features—2 features for identifying the presence of artifacts in these images and another 2 features for assessing its focus.

### 4.3. Acquired Images Focus Assessment Results

Finally, the acquired images were also evaluated in terms of image focus assessment. The classification results for the best model is presented in Table 7 (fourth row). Moreover, only two features were used for this task, namely the *Mean of the values of y of the Marziliano Metric of the gray image* ($PRCB_{I_{Gray}}^{MEAN_{Y}}$) and the *Difference between the sum of the values of the image curvature of both images* ($CURV_{I_{Gray}}^{SUM}-CURV_{I_{Blur}}^{SUM}$). As one can infer from these results, an overall classification accuracy of 86.2% was attained for image focus assessment.

### 4.4. Algorithm Running Times

Being real-time processing of the camera preview images an issue when dealing with a device with limited capabilities, the proposed pipeline running time for assessing a single image was studied. A low-end device (Nexus 5) and two high-end devices (Samsung S9 and OnePlus 6T) were selected for evaluation. Mean running time for preview and acquired image focus assessment is presented in Table 8.

## 5. Discussion

Acquiring skin lesion images using a smartphone is undoubtedly becoming more frequent in our daily lives, not only by professionals but also by patients seeking clinical guidance. By using handheld and decentralized image acquisition approaches, the time between the identification of a potential lesion and a diagnosis could be drastically shortened. However, if the quality of the acquired images is not assessed during or immediately after an acquisition, the number of unsuitable images for a clinical assessment may dramatically increase. Recent studies point out that professionals do not necessarily need to be trained photographers to ensure an adequate acquisition of quality images [18]. However, if some guidelines or even real-time guidance is provided to the user, the number of images with enough quality may increase significantly, reducing the number of times that an unsuitable picture is sent for analysis. Thus, image quality assessment solutions for dermoscopic image acquisition should be provided or adopted, in order to improve current dermatological screening processes.

With this clear goal in mind, the methodology proposed in this work was revealed to be suitable and robust enough to fulfill this purpose. In particular, accurate results were obtained during this study for the focus assessment of mobile-acquired skin mole dermoscopic images. Additionally, suitable results were also obtained for the detection of artifacts on dermoscopic images, as well as for the focus assessment on camera preview images. Remarkably, only two highly discriminating focus metrics are used to achieve the reported results in each classification task, thus making this approach suitable for real-time usage with mobile devices. Despite these results, when combining this two individual tasks, the overall performance of the proposed solution suffers a significant decrease, as expected. This loss in performance may be due to the inherent complexity of each task. Individual limitations for each type of assessment may be unveiled and more pronounced when looking into these two different tasks as one. Despite this decrease, the overall performance for focus assessment of preview images in terms of artifact detection and focus is quite satisfactory.

**Figure 4:**
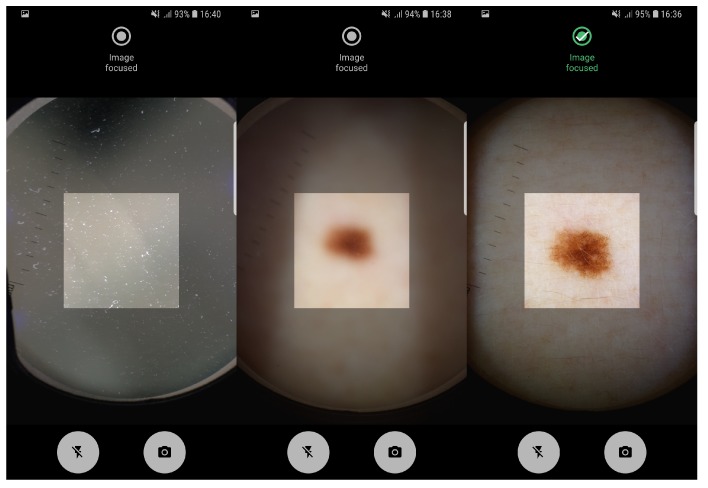
Application screenshots of: artifact detection module and real-time preview focus assessment indicating non-focused and focused image, respectively.

The proposed pipeline in this study, involving artifact detection, preview and taken image assessment was already deployed in an Android application running on a smartphone. The application allows the manual acquisition of dermoscopic images in an easy and intuitive way, providing real-time feedback about the level of focus of the images being acquired. In Figure 4 it is possible to observe the feedback provided by Preview Image Assessment module. Moreover, usability tests on the application interfaces design were already made and reported in Reference [31]. Additionally, the proposed solution was designed not only to evaluate the image quality of mobile-acquired dermoscopic images, but also the focus level of each preview frame in real-time, in order to guide the user during the acquisition process. Given the reported running times on both low-end and high-end devices, we can also conclude that the proposed approach is computationally suitable for real-time usage on mobile devices.

Thus, given the importance of such images for diagnosis purposes, we can consider that the ultimate goal of this study was fulfilled, since the process of acquiring dermoscopic images of skin moles can now be simplified and better quality images can be collected for screening and diagnosis purposes. Particularly, by embedding this image quality assessment methodology in handheld image acquisition tools, standardized images can be obtained, which may increase the efficiency in the dermatological clinical flow.

## 6. Conclusions

Due to the constant increase and demand for telemedicine solutions, more specifically for dermatological purposes, is clear that standardization of the image acquisition process is a crucial step. Image quality evaluation of the acquired image is necessary, as well as providing adequate guidance to the end-user in the image acquisition process. In this paper, we presented a solution that acts on these two premises, being able to guide the user in real-time during the acquisition process, as well as assessing whether a certain mobile-acquired dermoscopic image is properly focused. Particularly, the proposed solution was designed for assisting the process of collecting a skin mole image in real-time, by using any smartphone camera with a dermoscope attached.

In terms of the automated analysis of camera preview images, our approach was divided into two different tasks—real-time artifacts detection and real-time preview images focus assessment, with obtained accuracies of 97.3% and 83.7%, respectively. An accuracy rate of 77.9% was achieved for the complete camera preview focus assessment module, which included both previously refferred tasks. Regarding image quality assessment of the mobile-acquired picture, the results obtained also demonstrate the adequacy of the proposed methodology, being achieved an accuracy of 86.2% using an approach that only requires the extraction of two lightweight focus metrics.

To finalize, an embedded Android application with the proposed methodology was also developed, in order to test the viability of the proposed approach in a real life scenario. Empirically, the results obtained through the real-time usage of the developed application seem to be in line with the results obtained through the validation datasets. However, further testing in real clinical settings are required, in order to properly evaluate the performance and suitability of the proposed approach for screening and diagnosis purposes.

For future work, it would be valuable to have the dataset annotated by specialists and enhanced with more images of different subjects and types of skin lesions in order to increase the robustness of our solution. Regarding the features used, further research should be done in the search and optimization of features capable of being used in real-time. Finally, testing the pipeline used in a real live scenario would be of utmost importance in order to provide more concrete indicators of the quality of the work here described.

## Figures and Tables

**Table 1:** Specification of Dermlite DL1 and DL3 dermoscopes.

Dermoscope	Dermlite DL1	Dermlite DL3
Polarization	Polarized & Non Polarized	Polarized & Non Polarized
Lighting	4 White LEDs	18 White LEDs
	(polarized/non-polarized)	(12 polarized, 6 non-polarized)
Optics	15 mm diameter	25 mm diameter
Magnification	10×	10×
Spectrum Control	No	PigmentBoost™
10 mm Reticle	Yes	Yes
Smartphone compatibility	Yes	Yes
Standalone usability	Yes	Yes

**Table 2:** Detailed list of the smartphones used in this study. Additional camera-related details are also provided for each smartphone.

Smartphone	Camera Resolution	Camera Aperture
J5 (2016)	13 MP	f/1.9
LG G6	13 MP	f/1.8
Huawei Mate 10 Pro	12 MP	f/1.6
Motorola G5	13 MP	f/2.0
Nexus 5	8 MP	f/2.4
Nexus 5X	12.3 MP	f/2.0
OnePlus 5	16 MP	f/1.7
S5	16 MP	f/2.2
S6	16 MP	f/1.9
S7	12 MP	f/1.7
S8	12 MP	f/1.7

**Table 3:** Image type distribution in the *DermIQA* dataset.

Image	Focused Images	Non Focused Images	Total
Preview image	451	543	994
Acquired picture	440	545	985
Total	891	1088	1979

**Table 4:** Visual characteristics of the skin moles included in the *DermIQA* dataset.

Subject	Color	Hair	Size	Shape	Border
**s1**	Light Brown	Yes	Small	Circle	Regular
**s2**	Light Brown	Yes	Big	Oval	Regular
**s3**	Brown/Dark Brown	No	Medium	Irregular	Irregular
**s4**	Black	No	Medium	Circle	Regular
**s5**	Light Brown	No	Big	Oval	Regular
**s6**	Brown	Few hairs	Small	Irregular	Irregular
**s7**	Dark Brown	Few hairs	Medium	Circle	Regular
**s8**	Brown	Beard	Medium	Oval	Regular
**s9**	Light Brown	Yes	Small	Oval	Regular
**s10**	Light Brown and Brown	No	Medium	Irregular	Regular
**s11**	Dark Brown/Black	Beard	Medium	Oval	Regular
**s12**	Black	No	Small	Oval	Regular
**s13**	Light Brown	No	Big	Oval	Regular
**s14**	Light Brown	Yes	Big	Oval	Regular

**Table 5:** Summary of the features extracted for focus assessment. * Each metric value was calculated for $I_{Gray}$, $I_{Blur}$, difference and division of blur and gray images.

Group	Acronym	Feature Name	Extracted Metrics *
Gradient based	GRAE	Energy Image Gradient	Sum, mean, std, max
	GRAS	Squared Gradient	Sum, mean, std, max
	TENG	Tenengrad	Sum, mean, std, max, var
Laplacian based	LAPE	Energy of Laplacian	Sum, mean, std, max
	LAPSM	Sum Modified Laplacian	Sum, mean, std, max
	LAPD	Diagonal Laplacian	Sum, mean, std, max
	LAPV	Variance of Laplacian	Mean, std, max, var
	LAPG	Laplacian and Gaussian	Sum, mean, std, max
Statistical based	GLVA	Gray Level Variance	Sum, mean, std, min, max
	GLVN	Norm. Gray L. Variance	Normalized variances
	HISE	Histogram Entropy	Sum (R, G, B, gray)
	HISR	Histogram Range	Range (R, G, B, gray)
DCT/DFT	DCT	DCT	Sum, mean, std, min, max
	DFT	DFT	Sum, mean, std, min, max
Other principles	BREN	Brenner’s Measure	Sum, mean, std
	CURV	Image Curvature	Sum, mean, std, min, max
	SPFQ	Spatial Freq. Measure	Sum, mean, std, max
	VOLA	Vollath’s autocorrelation	Sum, mean, std, max
	PRCB	Perceptual blur	Sum and mean (x and y axis)

**Table 6:** Decision Tree tested hyper parameter values used during training for model optimization.

Hyperparameter	Search Space Values
Max Depth	1,2,3
Split Criterion	Gini, Entropy
Split Strategy At Each Node	Best, Random
Minimum Samples To Slip	1/3, 1/2 and 1/1 of the train size

**Table 7:** Classification results for best performer models.

Models	Accuracy (%)	Recall (%)	Precision (%)	Specificity (%)	F1-Score (%)
(1) Artifact detection	97.3	96.96	100	100	98.5
(2) Focus assessment	83.7	85.4	79.5	82.4	82.4
(1+2) Preview assessment	77.9	80.5	72.8	75.8	76.5
(3) Acquired Focus assessment	86.2	91.1	80.7	82.1	85.6

**Table 8:** Algorithm running times on different smartphones for preview and acquired images focus assessment.

Smartphone	Preview Image Assessment Speed (ms)	Acquired Image Assessment Speed (ms)
Nexus 5 (low end)	625	1203
Samsung S9 (high end)	77	126
OnePlus 6T (high end)	58	123
